# Geographic clustering and population structures of Campylobacter jejuni and Campylobacter coli in South and Southeast Asian poultry systems

**DOI:** 10.1099/mgen.0.001706

**Published:** 2026-05-07

**Authors:** Burhan Lehri, Le Thi Hong Nhung, Keya Ghosh, Tahia Ahmed Logno, Mahamudul Hasan, G. Weerasooriya, M.A.R. Priyantha, P.S. De Alwis, Venkateshprabhu Janagaraj, Zayina Zondervenni Manoharan, Sadiq Dantroliya, Ramesh Pandit, Chaitanya Joshi, Luu Quynh Huong, M. Anandachitra, Madhvi Joshi, Rashed Mahmud, Pham Thi Ngoc, Archibald Coombs, Ying Ying Chrystal Li, Mahati Ramachandra, Saira Butt, Samuel M Ronald, Anne Conan, Paritosh Kumar Biswas, Mohammed Abdus Samad, Ruwani Kalupahana, Sitara Swarna Rao Ajjampur, Guillaume Fournie, Brendan W. Wren, Fiona Tomley, Damer P. Blake, Richard A. Stabler

**Affiliations:** 1 Department of Infection Biology (DIB), London School of Hygiene and Tropical Medicine (LSHTM), London, UK; 2 National Institute of Veterinary Research (NIVR), Ha Noi, Vietnam; 3 Chattogram Veterinary and Animal Sciences University, Chittagong, Bangladesh; 4 Transboundary Animal Disease Research Center, Bangladesh Livestock Research Institute (BLRI), Savar, Bangladesh; 5 Veterinary Research Institute, Gannoruwa, Peradeniya, Sri Lanka; 6 The Wellcome Trust Research Laboratory, Division of Gastrointestinal Sciences, Christian Medical College, Vellore, India; 7 Gujarat Biotechnology Research Centre (GBRC), Department of Science and Technology (DST), Government of Gujarat, Gandhinagar 382011, India; 8 Tamil Nadu Veterinary and Animal Sciences University, Tamil Nadu, India; 9 Department of Pathobiology and Population Sciences, Royal Veterinary College, London, UK; 10 Centre for Applied One Health Research and Policy Advice (OHRP), City University of Hong Kong, Hong Kong, PR China; 11 ASTRE, CIRAD, INRAE, University of Montpellier, Montpellier, France; 12 Centre de Coopération Internationale en Recherche Agronomique pour le Développement (CIRAD), Harare, Zimbabwe; 13 Department of Veterinary Public Health & Pharmacology, University of Peradeniya, Peradeniya, Sri Lanka; 14 INRAE, VetAgro Sup, UMR EPIA, Université Clermont Auvergne, Saint-Gènes-Champanelle, Saint-Genès-Champanelle, France; 15 Department of Pathobiology and Population Sciences, Royal Veterinary College, Hawkshead Lane, Brookmans Park, Hatfield AL9 7TA, UK

**Keywords:** Asia, *Campylobacter coli*, *Campylobacter jejuni*, chicken, genomic diversity, whole-genome sequencing

## Abstract

Poultry production is rapidly expanding across South and Southeast Asia to meet rising demand for human consumption. *Campylobacter jejuni* and *Campylobacter coli* are the leading causes of human bacterial gastroenteritis worldwide, with poultry serving as a primary reservoir posing significant risks to food safety and public health. This study investigated the genetic diversity of *C. jejuni* and *C. coli* isolates from chicken sampled in farms, along with markets (live bird markets and shops) and slaughtering facilities from Bangladesh, India, Sri Lanka and Vietnam. Core-genome multilocus sequence typing revealed strong geographical clustering of *C. coli* isolates, dominated by ST-828 clonal complex lineages largely confined within national borders. In contrast, *C. jejuni* isolates clustered more diffusely into multiple clonal complexes (ST-21, ST-45 and ST-257) that were shared between countries, reflecting greater ecological adaptability and frequent gene flow. Permutational multivariate ANOVA (PERMANOVA) supported these patterns; geography explained more genomic variation for *C. coli* than *C. jejuni,* while poultry breed type and production site contributed little overall. An exception was Sri Lanka, where *C. jejuni* showed an apparent ecological segregation between markets and slaughtering facilities, albeit with a limited within-country sample size. Genomic SNP-based analysis highlighted recombination as a significant evolutionary force maintaining distinct country-specific lineages in *C. coli*, while supporting a broader geographic mixing of *C. jejuni*. Pangenome analyses were consistent with more geographically structured accessory profiles in *C. coli* and a more dispersed pattern in *C. jejuni*, compatible with broader regional dissemination. Comparison with global reference genomes showed these isolates clustered with strains from diverse host species, highlighting the ability of *C. jejuni* to occupy multiple ecological niches and spread across borders. The distinct patterns of spatially clustered and dispersed lineage distributions for *C. coli* and *C. jejuni*, respectively, highlight the importance of understanding species-specific transmission dynamics to inform targeted intervention strategies. This understanding underscores the need for interventions along poultry production and distribution networks that can effectively mitigate the risks associated with campylobacteriosis.

**Impact Statement**

This study provides the first large-scale comparative genomic analysis of *Campylobacter jejuni* and *Campylobacter coli* across multiple poultry production networks in South and Southeast Asia, revealing distinct species-specific transmission patterns with implications for future surveillance design programmes and risk mitigation strategies. The research demonstrates that *C. coli* populations exhibit strong geographical clustering with lineages largely confined within national borders, while *C. jejuni* shows greater ecological adaptability with transnational spread across regional boundaries. This fundamental difference in population structure and transmission dynamics highlights the need for tailored surveillance and control strategies for *Campylobacter* spp. As poultry production rapidly intensifies globally to meet growing demand, these insights into species-specific *Campylobacter* ecology fill a critical surveillance gap in low- and middle-income countries and provide an essential foundation for designing targeted interventions to mitigate foodborne disease burden.

## Data Summary

Whole-genome sequencing data are available under BioProject: PRJNA1224230. Isolate accession numbers are provided in Table S1. All computational tools are either publicly available or provided on https://github.com/Blehri/Campylobacter-population-genomics-asia.

## Introduction

The importance of food safety and security is rising globally, particularly in South and Southeast Asia, driven by rapid agricultural development and growing human populations. As demand for nutrition increases, poultry production that provides cost-effective and scalable sources of protein provision is intensifying [1]. However, the intensification of poultry farming has increased the risk of zoonotic transmission of bacterial pathogens such as *Salmonella* spp., *Campylobacter* spp. and *Escherichia coli. Campylobacter* is one of the most common causes of bacterial gastroenteritis worldwide, associated with 7.5 million disability-adjusted life years [2], with *Campylobacter jejuni* estimated to cause up to 75% of cases and *Campylobacter coli* up to 15% [3]. Many *Campylobacter* species have broad ecological plasticity, with the capacity to spread and survive in various host species, making them resilient pathogens that pose significant challenges for food safety [4]. This adaptability allows some *Campylobacter* spp. to thrive and spread in diverse ecological niches, including poultry farms and processing facilities, where cross-contamination can occur during production and handling processes [5].

Genetic comparisons between *C. jejuni* and *C. coli* have shown that both *C. jejuni* clonal complexes (CCs), CC ST-21, CC ST-45 and *C. coli* CC ST-828, have broad ecological plasticity [4], suggesting that both species have the potential for rapid adaptation to many ecological niches. *C. coli* isolates group into three main phylogenetic clades (clades 1–3), with clade 1 showing gene mixing from *C. jejuni* [6,7]. This interspecies gene flow is thought to be driven by agricultural intensification, underscoring a potential convergent evolutionary trajectory of *C. coli* and *C. jejuni*, particularly associated with high density of large poultry production units, where both species co-circulate [8,9] and genetic material has a higher likelihood of mixing due to housing large flocks in close proximity [6,9].

To date, few large-scale, comparative genomic studies have focused on isolates detected in production and distribution networks within low- and middle-income countries (LMICs). Most documented *Campylobacter* genomes originate from high-income countries (HICs) [10]. A study based on 883 *C*. *jejuni* genome sequences in the PathoSystems Resource Integration Center database included only India (91 isolates) and Bangladesh (6 isolates) LMICs from Asia; however, these were all of human origin [11]. A whole-genome sequencing (WGS) study of 112 isolates of *Campylobacter* from diarrhoea patients at 2 hospitals in Kolkata, West Bengal, identified 90 *C*. *jejuni*, 20 *C*. *coli* and 2 *Campylobacter lari* isolates [12]. The isolates were diverse, with *C. jejuni* consisting of 39 known and 7 novel sequence types (STs) and *C. coli* consisting of 8 known STs and 1 novel ST [12]. In *C. jejuni*, the most common ST was ST-2131, which has not been reported in other countries [12], demonstrating that the diversity in LMICs is not currently represented well in the databases. A smaller Thai study looked at 21 *C*. *jejuni* and 5 *C*. *coli* isolates from commercial broilers and native chickens, which again identified rare and unusual STs [13]. Isolated *Campylobacter* from chicken meat in Vietnamese retail found that *C. coli* was the most prevalent species (35/46 isolates) and comprised 15 STs, of which 4 were novel [14]. This highlights a major knowledge gap regarding the ecology, evolutionary and transmission dynamics of *C. jejuni* and *C. coli* in these settings. Our study was undertaken to address this gap and lay the groundwork for designing targeted interventions for mitigating the burden of *Campylobacter*-related disease.

Rapidly intensifying chicken production in LMICs is highly diverse in terms of raised chicken types, modes of production and distribution. It is, therefore, important to understand ecological interactions between the two bacterial species under different environmental conditions to evaluate their public health implications, so as to allow for better-informed risk mitigation strategies for managing antibiotic resistance that may arise from their interactions within shared environments. The current study analysed over 600 *C*. *jejuni* and *C. coli* isolates from chickens in farms and ‘endpoints’ (live bird markets and live bird shops grouped as markets, along with slaughtering facilities) across South (Bangladesh, India and Sri Lanka) and Southeast Asia (Vietnam), including from local breeds to high production exotic hybrids. This study assessed genetic variations of these isolates across different strata and contextualized the findings against global data. The findings provide insights into how *C. jejuni* and *C. coli* spread across sampled sites and are relevant for future surveillance design programmes and risk mitigation interventions.

## Methods

### Isolation

**Fig. 1.**
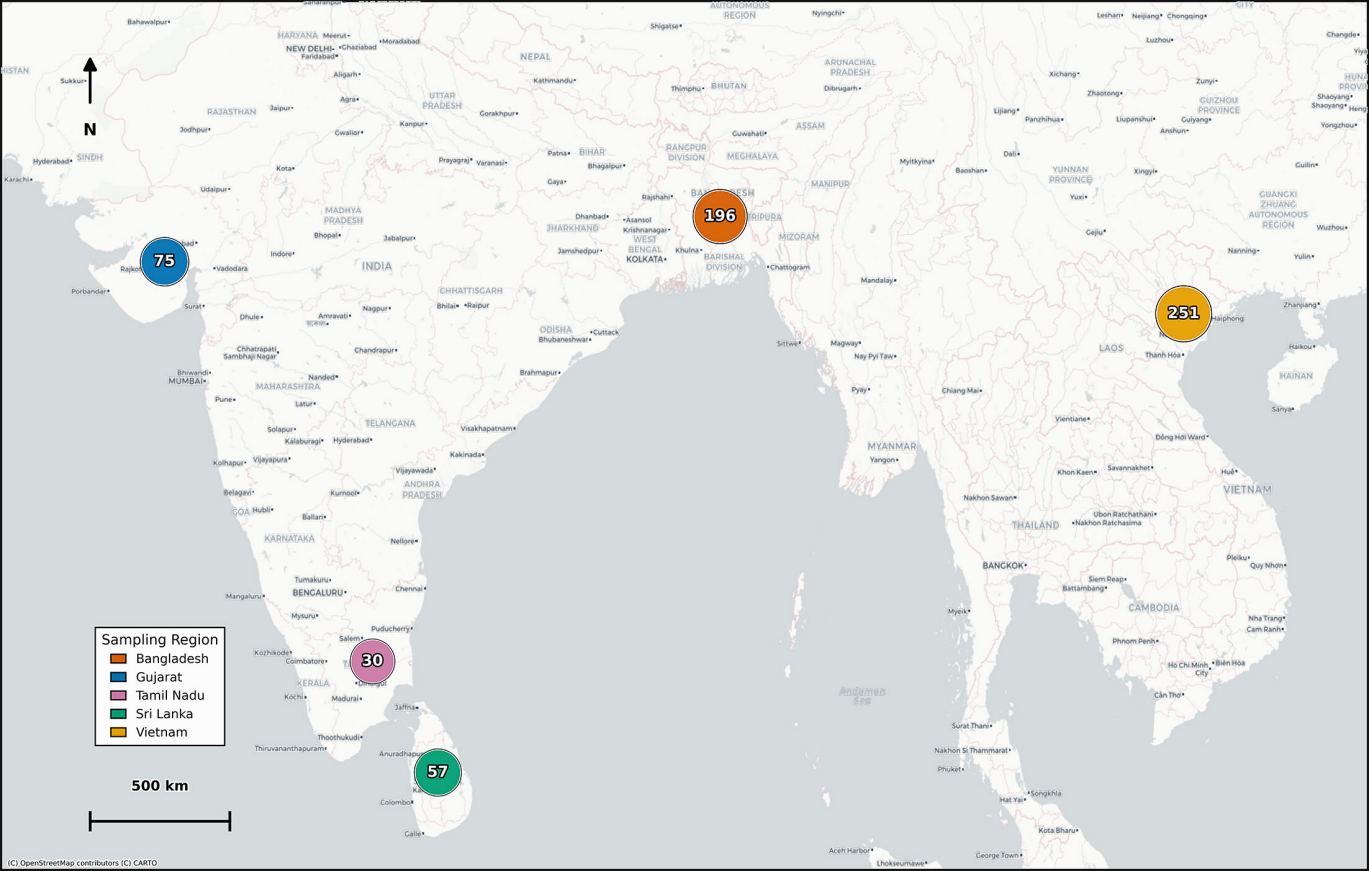
Map of isolation locations for *Campylobacter* isolates. Each marker represents the cumulative number of isolates collected from each site. Consensus locations are illustrated to maintain confidentiality.

*C. coli* and *C. jejuni* were isolated from chicken caeca sampled across production and distribution networks in Bangladesh, India, Sri Lanka and Vietnam, as part of a cross-sectional study conducted by the UKRI GCRF One Health Poultry Hub consortium [Fig. 1, Table S1 (available in the online Supplementary Material)]. The type of chickens sampled, the geographical coordinates and the type of sites were recorded (Table S1). In brief, the study tracked four chicken types, genetic hybrids, such as imported Exotic Broilers associated with short production cycles (30–45 days), along with slow-growing chickens Sonali and coloured feather hybrids raised for 70 days to several months and unique to Bangladesh and Vietnam, respectively. Along with Desi (‘native’) birds for Bangladesh, India and Sri Lanka, each having distinct local genetic backgrounds relative to their region. The study also recorded site types involved, such as farms, along with ‘endpoints’ markets (live bird markets and shops) and slaughtering facilities.

Isolation was in line with ISO 10272-1 : 2017 [15], with the addition of tazobactam (Merck) where necessary [16]. Isolation involved directly plating caecal content onto charcoal cefoperazone deoxycholate agar, with the addition of tazobactam (4–10 µg ml^−1^), followed by incubating for 48 h at 42 °C in containers with CampyGen (Oxoid) sachets to create microaerophilic conditions (10% CO_2_, 5% O_2_ and 85% N_2_). After which, growth was assessed for morphology, followed by selecting and plating five suspected *Campylobacter* spp. colonies on to Preston agar base, supplemented with *Campylobacter* Selective Supplement and 5% lysed horse blood (Oxoid). These isolates were further grown for 48 h at 42 °C under microaerophilic conditions. Initially, biochemical tests were used to rapidly screen isolates prior to PCR. Oxidase positivity was confirmed via colour change to blue/purple using oxidase strips (Merck) as per manufacturers' protocol, followed by hippurate discs (Thermo Scientific) as per manufacturers’ protocol to speciate *Campylobacter*, as per ISO 10272-1 : 2017 recommendation [15,17]. Further screening of single re-streaked isolates was conducted to minimize mixed colony sequencing using colony PCR following the conditions and primers for *C. coli* and *C. jejuni* identification as suggested by Wang G *et al*. [18]. Specifically, *C. jejuni* was identified using *hipO* gene primers: forward (ACTTCTTTATTGCTTGCTGC) and reverse (GCCACAACAAGTAAAGAAGC), with an expected product size of 323 bp. *C. coli* was identified using *glyA* gene primers: forward (GTAAAACCAAAGCTTATCGTG) and reverse (TCCAGCAATGTGTGCAATG), with a product size of 125 bp. The 23S rRNA gene was amplified using forward (TATACCGGTAAGGAGTGCTGGAG) and reverse (ATCAATTAACCTTCGAGCACCG) primers with a product size of 650 bp in multiplex [18]. Colonies that did not present the expected banding patterns were not sequenced. For isolates that were sequenced, the final species assignment used for downstream analysis was based on WGS data.

### Growth post-isolation

*C. coli* and *C. jejuni* were cultured from −80 °C stock on Preston agar base, supplemented with *Campylobacter* Selective Supplement and 5% lysed horse blood. Growth conditions for isolated samples were 37 °C within a variable atmosphere incubator (Don Whitley Scientific, UK) with microaerophilic conditions (10% CO_2_, 5% O_2_ and 85% N_2_). Isolates were incubated for 48 h followed by re-plating for an additional 24 h.

### Sequencing and assembly

DNA extraction was conducted using either the PureLink Genomic DNA Mini Extraction Kit (Invitrogen) or the QIAsymphony (QIAGEN), with the QIAsymphony DSP DNA mini kit (QIAGEN) following manufacturer guidelines. Qubit (Invitrogen) using the dsDNA BR assay kit (Invitrogen) was used to determine DNA concentration.

Samples were DNA sequenced by the Gujarat Biotechnology Research Centre, London School of Hygiene and Tropical Medicine (LSHTM), Novogene or MicrobesNG. The sequencing platform was the MiSeq sequencer (Illumina, USA) with the 2×250 bp kit v2, and samples were sequenced following the manufacturers’ guidelines. Selected samples were additionally sequenced at LSHTM using a MinION sequencer (Oxford Nanopore, UK), with an R10 flow cell (FLO-MIN111), following manufacturers’ guidelines.

FastQ paired-end reads were initially trimmed using Trimmomatic-0.39 [19] with the PE function (phred 33; LEADING:3, TRAILING:3, SLIDINGWINDOW:4 : 15, MINLEN:35). Read quality was then assessed using FastQC v0.11.9 [20], and samples were assembled using Unicycler v0.4.7 [21] with default settings. For MinION data, hybrid assembly was conducted combining Illumina paired-end reads and single-ended MinION reads using Unicycler v0.4.7 [21]. Contigs of minimum length 1 kbp were chosen and assessed for quality using Quast v5.0 [22], and further assembly completeness and contamination were assessed using CheckM [23]. CheckM and QUAST summary metrics for each isolate can be found in Table S1. An annotation for each isolate was conducted using PROKKA v1.14.6 [24].

### Data submission

Sequenced isolates were deposited in GenBank under BioProject PRJNA1224230 (Table S1). Genome assemblies and isolate records were also deposited in the PubMLST *Campylobacter jejuni/coli* database for isolates meeting quality and typing criteria; three assemblies with N50 <20 kbp (BM10205CS, BF62804CS, TL20108), along with isolates having no ST designation due to unreliable allelic profiles (Table S1), were not submitted to PubMLST.

### National Center For Biotechnology Information (NCBI) data download

Additional *C. coli* and *C. jejuni* data, along with respective metadata, were downloaded using ncbi-genome-download. Only complete *C. jejuni* and *C. coli* genomes that belonged to the RefSeq database were downloaded to ensure higher quality, well-curated isolates (Table S2).

### ST, multilocus sequence typing and clonal complex identification

STs were identified using the multilocus sequence typing (MLST) v2.23.0 tool [25]. Clonal complexes were identified on the PubMLST Query Tool against the PubMLST database by providing allelic and ST data for each *C. jejuni* and *C. coli* isolate. MLST data were further assessed with PHYLOVIZ [26] using goeBURST distance [27]. Loci with unique full-length novel allele, as determined by MLST v2.23.0 tool [25], were given a unique allelic number (Table S1). Novel STs were submitted to PubMLST and numbers assigned.

### Tree generation

Core-genome multilocus sequence typing (cgMLST) was conducted using chewBBACA v3.3.9 [28], with the ‘*C. jejuni* training file’, along with the ‘*C. jejuni* INNUENDO_wgMLST_2021-05-30T22_06_50_902917’ schema having 2,795 loci. cgMLST was determined by including loci in at least 95% of isolates. A phylogram was generated using GrapeTree version 2.2 [29] with minimum spanning tree (MST) V2 and visualized by Interactive Tree of Life (iTOL) [30]. Clusters were defined as discrete sub-branches in the radial MST that were separated from neighbouring groups by multiple edges (i.e. by several steps in allelic differences). Specifically, we grouped isolates connected by short allelic distances into clearly distinct clusters.

ClonalFrameML v1.13 [31] recombination corrected phylogenetic analysis was conducted for both *C. coli* and *C. jejuni* by selecting reference genomes *C. coli* FDAARGOS_735 and *C. jejuni* NCTC11168 for each respective tree. SNP calling was conducted using Snippy (v4.6.0) with assembled genomes [32]. A maximum-likelihood (ML) phylogenetic tree was generated using the core-genome multi-FASTA alignment generated by snippy-core on IQ-TREE (v2.4.0) [33]. Resulting ML trees served as inputs for ClonalFrameML (v1.13) [31], which was performed with 100 expectation maximization simulations to estimate recombination parameters. ClonalFrameML v1.13 [31] output was visualized in iTOL [30].

### Statistical analysis

For each *C. jejuni* and *C. coli* cluster, the proportion of isolates by country, city, production network site, poultry type, clonal complex and ST was quantified. For country, production network site and poultry type distributions, values were reported as observed percentage, expected percentage under a random-mix null model and enrichment ratio (observed ÷ expected). Diversity within clusters was summarized with the Shannon index (*H′*, higher values indicate greater diversity) and the Simpson index (1 − *D*, where values closer to 1 represent higher diversity).

To test whether a cluster’s country composition differed from the expected distribution, chi-square (*χ*²) goodness-of-fit tests were performed. Expected counts were derived from the global distribution of countries across the entire species dataset. For production network and poultry type analysis, expected distributions were calculated separately for each country to control for geographic effects. *χ*² tests were performed where expected counts met the minimum expected cell count of five. Baseline distributions for poultry type and site type analyses were calculated separately for *C. coli* and *C. jejuni* to ensure species-specific comparisons. Clusters where ≥90% of isolates originated from a single country were classified as country-specific. Multiple testing was controlled using Benjamini–Hochberg; false discovery rate *q*-values are reported alongside raw *P*-values. All analyses were implemented in Python (pandas 2.2.2, NumPy 1.26.4, SciPy 1.12.0) and visualized with matplotlib 3.9.0 and seaborn 0.13.2; scripts are available at https://github.com/Blehri/Campylobacter-population-genomics-asia.

To assess the contribution of geographic and production factors to genetic variation, permutational multivariate ANOVA (PERMANOVA) was conducted using two distance measures. Firstly, cgMLST allelic distances, where distance was measured as the proportion of loci with non-identical alleles among loci shared by each isolate pair; missing pairwise alleles were excluded. Secondly, patristic distances were extracted from recombination-corrected ClonalFrameML trees. Analyses were performed using the adonis2 function in the vegan package (v2.6.4) with 999 permutations (seed=42), evaluating country, poultry type and production network site. To control for geographic confounding, poultry type and site analyses were stratified by country, restricting permutations within national boundaries. *R*² values indicated the variance explained by each factor. Statistical significance was assessed at 0.05. Analyses required at least three isolates per distance matrix and two groups per factor. PERMANOVA was implemented in R (v4.3.0) using tidyverse (v2.0.0), ape (v5.8) and vegan (v2.6.4); scripts are available at https://github.com/Blehri/Campylobacter-population-genomics-asia.

### Pangenome analysis

Roary v 3.11.2 [34] was used for pangenome analysis with parameters (-e, --mafft) on GFF files consisting of both NCBI references and study sequences, split by species (*C. jejuni* and *C. coli*). The Roary gene_presence_absence.csv output was used to generate a binary principal component matrix with Pagoo (v 0.3.17) [35] (Tables S3 and S4). Principal coordinate analysis (PCoA) was conducted by applying Jaccard distance on R to visually assess clusters of isolates (e.g. country, host source); 95% confidence ellipses around datapoints were overlaid using ‘stat_ellipse’, assuming a multivariate normal distribution of ordination coordinates.

### Map generation

Maps of sequenced isolates sampling location were created with Python with matplotlib and pandas for data handling. For confidentiality, latitude/longitude coordinates were replaced with regional centroids to show the general sampling location.

## Results

### Isolate characteristics

**Fig. 2.**
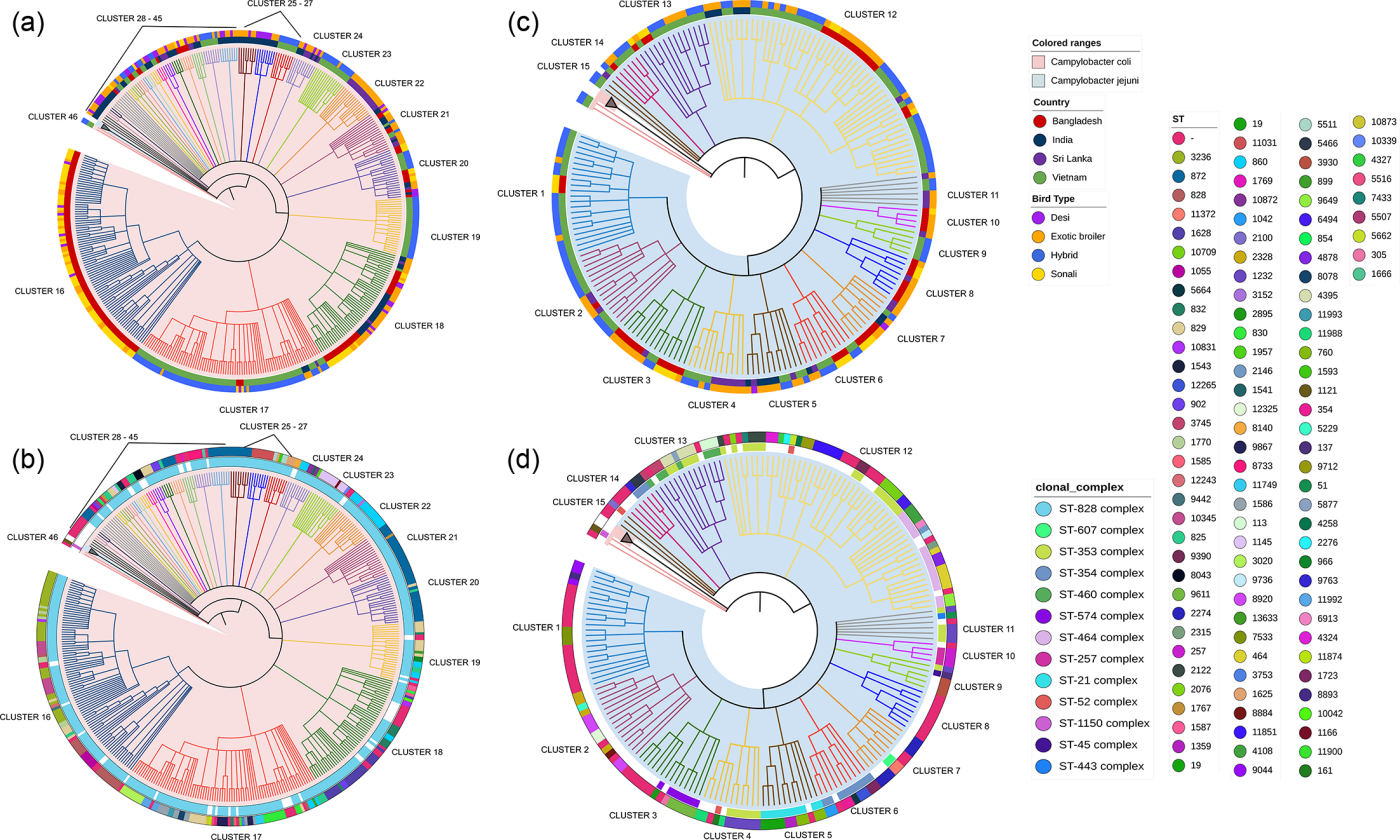
MST of *C. coli* and *C. jejuni* isolates from South (India, Sri Lanka and Bangladesh) and Southeast (Vietnam) Asia. CgMLST MST derived using chewBBACA and visualized on iTOL. (a and b) *C. coli* and (c and d) *C. jejuni*. (a and c) Concentric rings indicate a country of origin (inner ring) and chicken type (outer ring). (b and d) Concentric ring indicates clonal complex (inner ring) and ST (outer ring) for each isolate. Coloured branches define clusters across the tree.

Initially, isolation produced a higher proportion of samples of *C. coli* as opposed to *C. jejuni*; however, the number of *C. jejuni* isolates from each sample increased once extended-spectrum beta-lactamase (ESBL) *E. coli* contaminants were inhibited, with the addition of tazobactam to the isolation protocol [16]. In total, 616 *Campylobacter* spp. (*C. jejuni n=*194; *C. coli n=*422) were received and 609 (*C. jejuni n=*192; *C. coli n=*417) high-quality genome assemblies were produced (Table S1). CheckM indicated high overall assembly quality: 583/609 assemblies had ≥99% completeness and 579/609 had ≤1% contamination. Only seven assemblies showed contamination >5%; two of these did not yield a definitive ST assignment due to ambiguous or incomplete MLST allele profiles (Table S1). During isolation, there were instances where a single chicken sample yielded both *C. jejuni* and *C. coli* from the same host. For example, sample LM02202Cs, where LM02202Cs_s217_31 was *C. jejuni* and LM02202Cs_s216_31 was *C. coli* (Table S1). However, as the primary study objective was comparative genomic analysis rather than characterizing mixed-species colonization, isolation protocols were not optimized to detect all instances of mixed-species colonization in order to manage sample processing and sequencing capacity. The final species assignment was based on WGS data using MLST and phylogenetic inference. PCR-based speciation showed broad concordance with WGS-based assignment; for example, in Bangladesh, 181/196 (91.8%) isolates yielded a species-level PCR prediction, of which 34/40 (85%) *C*. *jejuni* and 126/140 (90%) *C*. *coli* isolates were correctly identified. A single *C. coli* was PCR positive for the *C. coli* amplicon, but without a *Campylobacter* species amplicon. The remaining isolates were misidentified as the other species. Four isolates produced only genus-level amplicons and nine were PCR negative. *Campylobacter* isolates were from Vietnam (251 isolates; 148 *C*. *coli* and 103 *C*. *jejuni*), Bangladesh (196; 144 and 52), India (105; 91 and 14) and Sri Lanka (57; 34 and 23) (Table S1). Mean genome size was 1.75 Mbp for *C. coli* (range 1.58–2.05 Mbp) and 1.71 Mbp for *C. jejuni* (range 1.58–2.02 Mbp). Overall, MLST analysis identified 111 different STs from 13 clonal complexes, plus 108 isolates without a known ST (including 74 novel allele combinations and 34 with at least 1 novel allele) (Table S1). A cgMLST MST identified 46 clusters, of which 31 belonged to *C. coli* (labelled clusters 16–46) and 15 belonged to *C. jejuni* (labelled clusters 1–15) (Fig. 2).

### Country distributions

As the number of isolates was not numerically identical across each of the four countries, a random distribution of isolates would give an expected distribution of 34.5% Bangladeshi, 35.5% Vietnamese, 21.8% Indian and 8.2% Sri Lankan isolates within a *C. coli* cluster and 53.6% Vietnamese, 27.1% Bangladeshi, 12.0% Sri Lankan and 7.3% Indian isolates for *C. jejuni*.

Analysis of *C. coli* clusters confirmed pronounced geographical structure (clusters 16/17 sizeable clusters *χ*², *P*<0.05; 71% of all *C. coli* genomes fell into ≥90% single-country clusters) (Table 1, Table S5_1). For instance, cluster 16 (*χ*², *P*=2×10⁻^36^) was entirely Bangladeshi (89/89, 100%), cluster 17 predominantly Vietnamese (72/79, 91%, *χ*², *P*=2×10^−23^), cluster 19 all Vietnamese (24/24, 100%, *χ*², *P*=1×10^−9^), cluster 22 all Sri Lankan (16/16, 100%, *χ*², *P*=8×10^−39^) and cluster 23 all Vietnamese (14/14, 100%, *χ*², *P*=1×10^−5^) (Table S5_1). Cluster 18 was enriched (*χ*², *P*=2×10^−7^) for Bangladeshi (27/53, 51%) and Indian (23/53, 43%) isolates. Among the smaller groups, clusters 26–28 were entirely Indian (8/8, 100% each). By contrast, mixed clusters, such as cluster 20 (Bangladeshi 10/21, 48%; Vietnamese 7/21, 33%; Indian 3/21, 14%; Sri Lankan 1/21, 5%; *χ*², *P*=0.59) and cluster 21 (Bangladeshi 4/17, 24%; Indian 7/17, 41%; Sri Lankan 3/17, 18%; Vietnamese 3/17, 18%, *χ*², *P*=0.075), showed no significant departure from the random expectation, illustrating that a minority of *C. coli* groups escape country specificity (Table S5_1).

**Table 1.** Country linkage to clusters. Core-genome clustering demonstrates a greater propensity for country linkage for *C. coli* than for *C. jejuni*. Cluster, cgMLST clusters (Fig. 2); isolates, number of isolates within cluster; >90%, clusters where a single country is prevalent in >90% of isolates. % indicates the proportion of clusters by country of isolation

	*C. jejuni*											
Cluster	1	2	3	4	5	6	7	8	9	10	11	12
Isolates	22	15	14	11	11	10	10	8	5	4	6	53
Vietnam	86%	73%	14%	45%	36%	50%	10%	0%	80%	0%	33%	60%
Bangladesh	14%	7%	79%	0%	0%	30%	50%	88%	0%	100%	0%	26%
Sri Lanka	0%	20%	7%	55%	18%	10%	20%	13%	20%	0%	67%	2%
India	0%	0%	0%	0%	45%	10%	20%	0%	0%	0%	0%	11%
>90%										Yes		
	***C. jejuni***			***C. coli***								
Cluster	13	14	15	16	17	18	19	20	21	22	23	24
Isolates	14	6	3	89	79	53	24	21	17	16	14	10
Vietnam	79%	83%	67%	0%	91%	2%	100%	33%	18%	0%	100%	0%
Bangladesh	21%	17%	0%	100%	4%	51%	0%	48%	24%	0%	0%	0%
Sri Lanka	0%	0%	33%	0%	1%	4%	0%	5%	18%	100%	0%	50%
India	0%	0%	0%	0%	4%	43%	0%	14%	41%	0%	0%	50%
>90%				Yes	Yes		Yes			Yes	Yes	
	***C. coli***											
Cluster	25	26	27	28	29	30	31	32	33	34	35	36
Isolates	8	8	8	8	7	5	5	4	4	3	3	2
Vietnam	100%	0%	0%	0%	100%	0%	0%	0%	0%	67%	0%	100%
Bangladesh	0%	0%	0%	0%	0%	0%	100%	0%	0%	33%	0%	0%
Sri Lanka	0%	0%	0%	0%	0%	40%	0%	0%	75%	0%	0%	0%
India	0%	100%	100%	100%	0%	60%	0%	100%	25%	0%	100%	0%
>90%	Yes	Yes	Yes	Yes	Yes		Yes	Yes			Yes	Yes
	***C. coli***											
Cluster	37	38	39	40	41	42	43	44	45	46		
Isolates	2	2	2	2	2	9	2	2	4	2		
Vietnam	0%	100%	0%	100%	0%	22%	0%	0%	0%	100%		
Bangladesh	100%	0%	100%	0%	0%	11%	0%	0%	0%	0%		
Sri Lanka	0%	0%	0%	0%	0%	11%	0%	0%	0%	0%		
India	0%	0%	0%	0%	100%	56%	100%	100%	100%	0%		
>90%	Yes	Yes	Yes	Yes	Yes		Yes	Yes	Yes	Yes		

Contrary to *C. coli*, the majority of *C. jejuni* clusters involved more than one country. Applying the ≥90% ‘country-specific’ threshold identified only one cluster (cluster 10); consequently, just 4 of the 192 *C*. *jejuni* genomes (2.1%) of the entire collection fell into a single country cluster (Tables 1 and S6_1). The remaining clusters consisted of isolates from across the study, for example, cluster 2 (*n* = 15) comprised Vietnamese (11/15, 73%), Sri Lankan (3/15, 20%) and Bangladeshi (1/15, 7%) isolates; cluster 3 (*n* = 14) was mostly Bangladeshi (11/14, 79%) with Sri Lankan (1/14, 7%) and Vietnamese (2/14, 14%) genomes. Even clusters dominated by one nation rarely exceeded the 90% cutoff. For example, cluster 13 was mostly Vietnamese (11/14, 79%), while cluster 12 (*n* = 53) mirrored the expected random mix (Vietnamese 60%, Bangladeshi 26%, Indian 11% and Sri Lankan 2%). Thus, despite the larger numbers of Vietnamese and Bangladeshi isolates in the dataset (Table S1), *C. jejuni* maintains a broadly intermingled geographic distribution, in stark contrast to the pronounced country-restricted clustering observed for *C. coli*.

### Poultry type associations

The dataset comprised 50 Desi, 214 Exotic Broiler, 230 Coloured Feather Hybrid and 114 Sonali isolates (Desi/Exotic/Sonali from India, Bangladesh and Sri Lanka; Coloured Feather Hybrid exclusively from Vietnam).

For *C. coli*, poultry type patterns were largely confounded by the strong geographic structure. To remove this bias, we first calculated, within each country, the overall poultry mix that served as a null baseline: Bangladesh, Sonali 64%, Exotic Broiler 24%, Desi 13% (*n* = 144); Vietnam, Coloured Feather Hybrid 95%, Exotic Broiler 5% (*n* = 148); India, Exotic Broiler 67%, Desi 33% (*n* = 91); and Sri Lanka, Exotic Broiler 100% (*n* = 34) (Table S5_3).

After testing the cluster against the species-specific baseline of its own country, no *C. coli* clusters showed poultry breed type distributions that significantly deviated from country-specific expectations (*χ*², *P*>0.05), showing that *C. coli* populations mainly reflect local breeding practices rather than breed-specific clustering. For example, cluster 16 (*n* = 89; *χ*², *P*=0.24) entirely Bangladeshi comprised Sonali 62/89 (69.7%), Exotic Broiler 21/89 (23.6%) and Desi 6/89 (6.7%) isolates, matching Bangladeshi *C. coli* baseline (Table S6_3).

Mixed clusters nevertheless reflected regional breeding preferences. In cluster 18 (*n* = 53; *χ*², *P*=0.23), the poultry type breakdown was Desi 7/53 (13%), Exotic Broiler 31/53 (58.5%) and Sonali 15/53 (28.3%) (Table S5_3). The geographic distribution within cluster 18 (Bangladesh 27, India 23, Sri Lanka 2 and Vietnam 1) revealed that Bangladeshi isolates leaned towards Sonali/Desi, whereas Indian and Vietnamese isolates were predominantly Exotic Broiler, again mirroring local husbandry practices (Table S6_3). Taken together, these distributions show that most *C. coli* populations mirror the country-specific portfolio of poultry types rather than exhibiting a breed-driven clustering pattern. A comparable tendency emerged for *C. jejuni*, albeit with weaker influence from country clustering, with country-specific baselines of Bangladesh, Exotic Broiler 58% and Sonali 42% (*n*=52); India Exotic Broiler 93% and Desi 7% (*n*=14); Sri Lanka Exotic Broiler 91% and Desi 4% (*n*=23); and Vietnam Coloured Feather Hybrid 86% and Exotic Broiler 14% (*n*=103) (Table S6_3), consistent with the broader geographic mixing described above.

### Cluster-level observations, geographic structure and ST complexes

In *C. coli*, the cgMLST-defined clusters were predominantly country-specific, although a few showed broader distribution. For instance, the most common *C. coli* ST was ST-872 (*n=*60), found in every country (Bangladesh 19, India 29, Sri Lanka 4 and Vietnam 8), across both farm and food endpoint sites, and in all poultry types (Desi 13, Exotic Broiler 29, Coloured Feather Hybrid 7 and Sonali 11) (Table S1, Fig. 2b).

ST-872 appeared in multiple clusters, including the pan-country cluster 21 (16/17 isolates), which consisted of isolates from Bangladesh (4/17, 24%), India (7/17, 41%), Sri Lanka (3/17, 18%) and Vietnam (3/17, 18%), as well as clusters 20, 27, 28, 31 and 42, showing that a single ST can arise in distinct phylogenetic contexts.

Other *C. coli* populations exhibited strong localized expansions. Cluster 16 in Bangladesh (89/89, 100%) was dominated by ST-3236 (34/89, 38%), which for the studied samples was unique to Bangladesh, alongside ST-828 (17/89, 19%) of which 17/18 was identified in Bangladesh, ST-1770 (7/89, 8%), and smaller sublineages (Table S1, Fig. 2a). Cluster 17 (*n=*79, 91% Vietnamese) contained a broad ST mix, e.g. ST-1586 and ST-830 (each 10/79, 13%), ST-829 (7/79, 9%), ST-3020 (9/79, 11%), ST-9867 (6/79, 8%) and several unknowns reflecting multiple, country-specific sublineages in Vietnam. Further examples of localized lineages included cluster 19 (*n=*24, predominantly Vietnamese), which contained ST-829 (9/24, 38%) and ST-860 (5/24, 21%), and cluster 22 (*n=*16, all from Sri Lanka), dominated by ST-860. These expansions illustrate how particular STs (e.g. ST-860) can characterize discrete, country-specific clusters in *C. coli* (Fig. 2b, Table S1).

For *C. jejuni*, established clonal complexes (e.g. ST-21, ST-257, ST-464 and ST-574) drove the branch structure in the study, only clustering (Fig. 2d, Table S1). CC ST-21 isolates occupied cluster 5 (10/10), CC ST-257 cluster 10 (3/3), CC ST-464 cluster 12 (15/15) and CC ST-574 in cluster 3 (7/7, 50%) (Table S1). The most common *C. jejuni* ST, ST-1232 (*n=*11, all Sri Lankan), belonged to CC ST-353 complex, which included 30 isolates from Bangladesh, India, Vietnam and Sri Lanka, mainly from Exotic Broilers (25/30, 83%) (Table S1).

Altogether, these results showed how geography and cluster-level clonal complex composition shape *C. coli* and *C. jejuni* diversity in South and Southeast Asia. *C. coli* typically displayed highly local expansions dominated by CC ST-828 complex (with occasional separate lineages, e.g. unknown CC STs and two CC ST-1150), whereas *C. jejuni* exhibited more heterogeneous complexes. The recurring appearance of certain STs (e.g. ST-872 and ST-829) across multiple clusters and countries points to both local expansions (e.g. cluster 16 in Bangladesh) and pan-regional sublineages that can traverse geographic or ecological boundaries.

### Production processing site

Overall, the total distribution by country and processing site type for both *Campylobacter* species included Bangladesh (farm=132, market=64), India (farm=34, market=71), Sri Lanka (market=37, slaughtering facility=19) and Vietnam (farm=133, market=49, slaughtering facility=69) isolates (Table S1).

*C. coli* (*n* = 417) was recovered from farms (186, 45%), markets (172, 41%) and slaughtering facilities (59, 14%). Country-specific *χ*² tests showed that cluster site distributions reflected country-specific sampling patterns (1/16 clusters showed *P*<0.05; cluster 18 *P*=0.018), backing the idea of a farm-to-slaughter progression of isolates (Table S5_2). Cluster 16 (*n* = 89) contained 57% farm (51/89) vs. 43% market (38/89) isolates (Table S5_2), whereas cluster 17 (*n* = 79, of which 72 from Vietnam) included 54% farm (39/72), 17% market (12/72) and 29% slaughtering facility (21/72) reflecting country-specific practice (Table S5_2).

*C. jejuni* was primarily recovered from farms, with 113/192 genomes (59%) from farms, 49/192 (26%) from markets and 29/192 (15%) from slaughtering facilities (Table S6_2). Across clusters, farm isolates usually made up ~50–65%, market ~25–30% and slaughtering facilities ~10–20%. Cluster 12 (largest, *n* = 53) mirrored this global mix 64% farm (34/53), 26% market (14/53) and 9% slaughtering facility (5/53) (Table S6_2). At the extremes, cluster 11 (*n* = 6) was 83% slaughtering facility (5/6) with no farm genomes, whereas cluster 8 (*n* = 8) was 88% farm and only 13% market. A ‘balanced’ example is cluster 1 (*n* = 22; predominantly Vietnamese, 19/22 isolates): 58% farm (11/19), 26% (5/19) market and 14% (3/19) slaughtering facility, again matching the overall ratio (Table S6). These patterns indicate *C. jejuni* bacteria movement along the farm-to-fork chain, emerging on farms, mixing at markets and persisting into slaughtering facilities.

### ClonalFrameML analysis relative to cgMLST results

Homologous recombination in *Campylobacter* is known to generate genome diversity and may confound phylogenetic inference if unaccounted for [36,37]. Thus, to assess the impact of recombination on phylogenetic relationships, ClonalFrameML analysis was conducted on both *C. coli* (Fig. 1 SA–B) and *C. jejuni* isolates (Fig. 1 SC–D). Overall, the ClonalFrameML inferences were largely congruent with the cgMLST-defined clusters (Fig. S1B, D), supporting the geographic structuring observed in *C. coli* (Fig. S1A) and the relatively broader regional mixing in *C. jejuni* (Fig. S1C). For *C. coli*, the recombination-corrected phylogenies confirmed that some clusters were highly country specific (e.g. exclusively Bangladeshi or Vietnamese) (Fig. S1A), mirroring the tight grouping seen in cgMLST. By contrast, *C. jejuni* remained less segregated geographically in both cgMLST (Fig. 2c) and ClonalFrameML (Fig. S1C) analyses, with numerous clusters encompassing isolates from multiple countries. Poultry type associations also followed a similar pattern in both analyses: *C. coli* clusters largely tracked a country’s preferred chicken lines (e.g. Sonali in Bangladesh, Coloured Feather Hybrid in Vietnam) (Fig. S1A), whereas *C. jejuni* showed more interspersed bird types (Fig. S1D). These findings indicate that while cgMLST captured most population-level structure, the ClonalFrameML analysis provided additional evidence that these geographic patterns reflect clonal descent rather than recombination artefacts.

### PERMANOVA analysis

PERMANOVA quantified the contribution of geography and production factors to genomic variation using two distance measures: cgMLST allelic distances and patristic distances from recombination-corrected ClonalFrameML trees. In *C. coli*, country accounted for 14.6% of variance with cgMLST (*R*²=0.146, *P*=0.001) and 16.9% with phylogenetic distances (*R*²=0.169, *P*=0.001). In *C. jejuni*, the country effect was weaker: 7.8% (cgMLST; *R*²=0.078, *P*=0.001) and 5.2% (phylogenetic; *R*²=0.052, *P*=0.001). Within-country effects were modest but revealed species-specific patterns. For *C. coli*, effects were weak and inconsistent: in Bangladesh, poultry type explained 2.6% of the variance by cgMLST (*R*²=0.026, *P*=0.039) but was not significant using phylogenetic distances (*R*²=0.027, *P*=0.058). A site effect was detected for *C. coli* in Bangladesh using phylogenetic distances only (*R*²=0.017, *P*=0.047). For *C. jejuni*, poultry type effects were more consistent, with Bangladesh for both distance measures (cgMLST *R*²=0.048, *P*=0.005; phylogenetic *R*²=0.046, *P*=0.039), in Sri Lanka by cgMLST (*R*²=0.091, *P*=0.047; phylogenetic *R*²=0.115, *P*=0.099) and in Vietnam by cgMLST (*R*²=0.019, *P*=0.014). Notably, *C. jejuni* in Sri Lanka showed a strong site type effect that exceeded the overall country effect: 21.7% of the variance by cgMLST (*R*²=0.217, *P*=0.002) and 18.6% by phylogenetic distances (*R*²=0.186, *P*=0.002). This pattern was unique to *C. jejuni* in Sri Lanka, where sampling encompassed only markets and slaughtering facilities (no farm samples), suggesting pronounced ecological differentiation, although with a sample size of 22, of which 14 were isolated from markets and 8 from slaughtering facilities. Together, these results corroborate the cluster-based patterns with stronger country structuring in *C. coli* and weaker country-level differentiation in *C. jejuni*, with modest, context-specific poultry type differences appearing more consistently for *C. jejuni* across multiple countries.

### Expanded MST perspective

To place the study isolates into a global context, a supplemental reference dataset of 373 isolates (300 *C*. *jejuni* and 72 *C*. *coli*) was added to the original cgMLST dataset (Table S2, Fig. S2). Globally, the distribution of *C. coli* is dominated by CC ST-828 sublineages, which also dominated samples from Bangladesh, India, Sri Lanka and Vietnam, showing the broad geographic range of these lineages (Fig. S2, Table S2). Similarly, a few *C. coli* CC ST-1150 isolates appeared in both local (e.g. Vietnam) and global datasets, indicating that this more recently recognized lineage also circulates outside Asia.

Some sequence types within these complexes appear exclusive to specific countries in this dataset, whereas others show wider geographic ranges (Table S2). For instance, ST-1359 (CC ST-21) was found only in India (two poultry isolates), whereas ST-760 (also CC ST-21) occurred in Taiwan (two human isolates) and Vietnam (four poultry isolates), and ST-19 (CC ST-21) spanned India (three poultry isolates), Sri Lanka (one poultry isolate) and Sweden (six isolates from *Gallus gallus* or *Bos taurus*). In addition, ST-137 (CC ST-45) was identified in Vietnam (one poultry isolate) as well as in Finland, the UK and the USA. These patterns highlight both localized population structure (consistent with local expansion) and transregional dissemination, likely driven by trade or human travel. Notably, the global dataset includes large clusters from countries such as Finland (e.g. *C. jejuni* ‘cluster 2’ at ~76%), yet the same clonal complexes were also observed in South Asian poultry (Table S2).

### Pangenome results

**Fig. 3.**
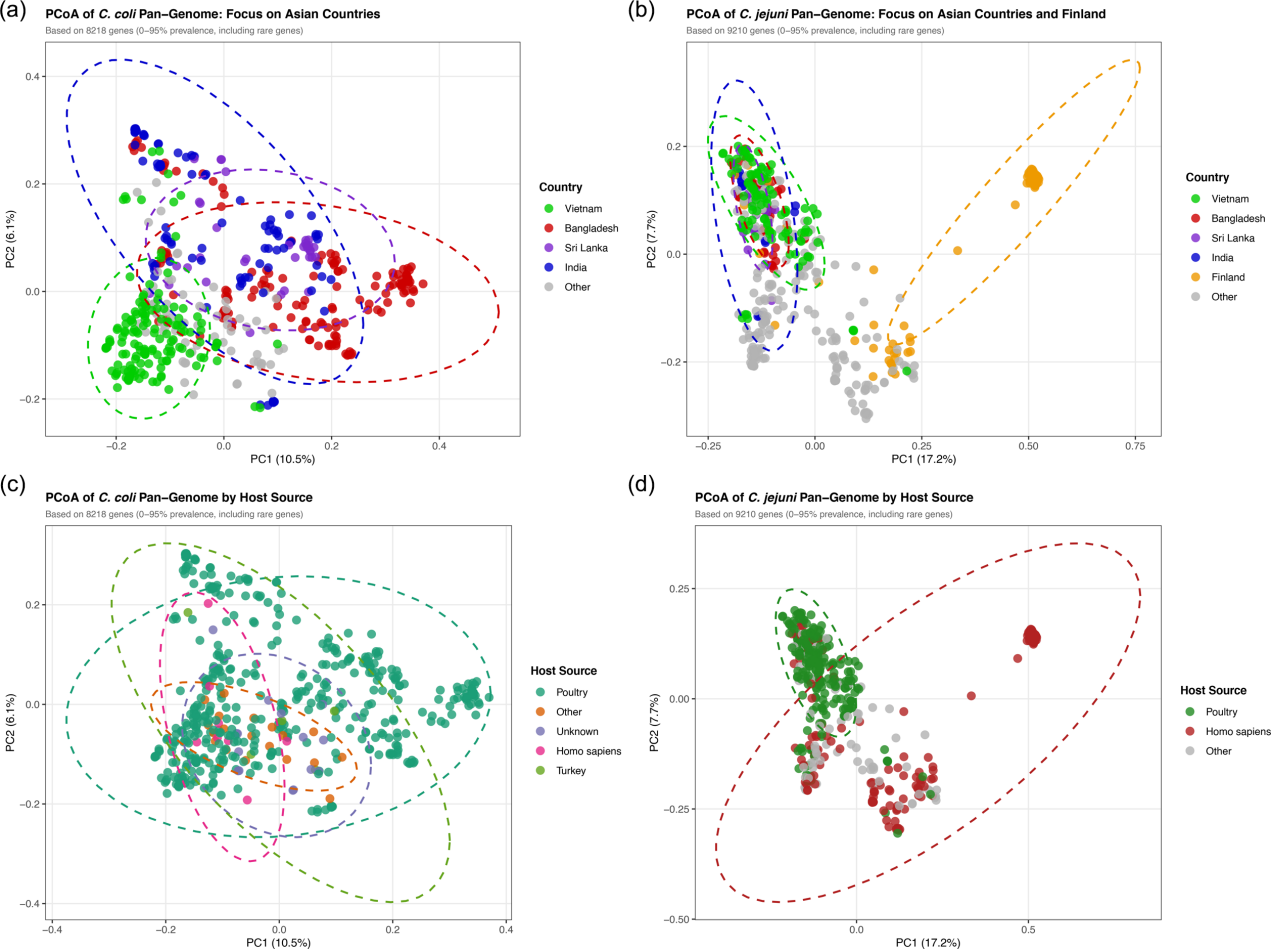
Analysis of accessory genes categorized by country. *C. coli* (a and c) and *C. jejuni* (b and d) PCoA based on the Jaccard distance from Roary outputs. Each point corresponds to an individual isolate plotted by the first two principal coordinates (PC1, PC2). The ellipses denote 95% confidence intervals around the isolates. (a and b) Isolates coloured by country: Bangladesh (red), India (blue), Sri Lanka (violet), Vietnam (green) and Finland (orange), while grey points are publicly available RefSeq genomes included for broader comparison. (c) *C. coli* isolates coloured by host: poultry (green), *Homo sapiens* (red), other (orange), Turkey (light green) and unknown (grey). (d) *C. jejuni* isolates coloured by host: poultry (green), *Homo sapiens* (red) and others (grey).

Accessory gene PCoA analysis was used to determine whether the presence/absence of variable gene sets could identify additional patterns, for example, country-specific genotypes, as opposed to the more stable core genome. *C. coli* pangenome consisted of 9,488 genes, of which 1,270 (13.4%) were core, 1,870 (19.7%) were shell genes (5–95%) and 6,348 (66.9%) were cloud genes (<5%). *C. jejuni* pangenome comprised 10,359 genes, including 1,149 (11.1%) core, 2,062 (19.9%) shell and 7,148 (69.0%) cloud genes. For downstream analyses, genes present in ≤95% of isolates were defined as accessory genes (Fig. S3). *C. coli* isolates from Bangladesh, India, Sri Lanka and Vietnam showed separation on the PCoA plot, with overlaps between countries (Fig. 3a). Vietnam formed a tighter group, as did Bangladesh, whereas India and Sri Lanka overlapped along PC1, forming a continuum positioned between Vietnam and Bangladesh (Fig. 3a). In contrast, the *C. jejuni* PCoA did not display country-specific grouping, except for Finland isolates (Fig. 3b), and showed separation between poultry and human isolated samples (Fig. 3d). *C. coli* did not show host-based separation, likely due to the large bias of poultry isolates compared to other isolation sources (Fig. 3c).

Overall, *C. coli* accessory gene patterns reflected the geographic structure observed in the cgMLST analysis, while *C. jejuni* exhibited less country-level separation except where an outbreak context (Finland) produced a distinct cluster. Both species showed partial mixing of host sources; however, *C. jejuni* displayed a clearer split between poultry and human isolates compared to *C. coli*, which showed broader overlap in its accessory genome profiles.

## Discussion

This study provides the first large-scale comparative genomic analysis of *C. jejuni* and *C. coli* (over 600 strains) across multiple poultry production networks in South Asia (Bangladesh, India and Sri Lanka) and Southeast Asia (Vietnam), revealing distinct species-specific transmission patterns, with implications for regional surveillance and intervention strategies. The isolation methods adopted led to a higher number of *C. coli* compared to *C. jejuni* (422 vs. 194), in contrast to numerous earlier studies often conducted in HICs where *C. jejuni* is primarily isolated [38]. In Bangladesh, high levels of ESBL *E. coli* may have influenced the detection of *C. coli* compared to *C. jejuni* isolates, necessitating the use of tazobactam as an additional selective agent [39].

A further limitation of this study is that, in most cases, only a single colony per sample was sequenced. Poultry can be simultaneously co-colonized by both *C. jejuni* and *C. coli*, as well as multiple strains, meaning that the single-colony approach underestimated mixed-species carriage and within-host lineage diversity. It has been estimated that up to 87 isolates/chicken would be required to detect 95% of the observed ST diversity [40]. Thus, the population structure described here likely reflects the dominant recovered colony from each sample rather than the full *Campylobacter* population present within individual birds. Nevertheless, the contrasting patterns observed for *C. coli* and *C. jejuni* were consistent across cgMLST, ClonalFrameML and PERMANOVA, suggesting that this limitation is unlikely to overturn the main between-species patterns reported. Sampling intensity varied across countries, sites and time periods (Fig. 1). Vietnam contributed 251 isolates, Bangladesh 196, India 105 and Sri Lanka 57, with site type representation differing by country; for example, Sri Lanka samples were exclusively obtained from markets and slaughtering facilities, with no farm sampling. In addition, some locations were sampled over relatively short time windows, limiting temporal resolution. This heterogeneity reflects logistical and cost constraints inherent to conducting coordinated, multi-country surveillance across diverse poultry production systems. These imbalances may reduce sensitivity for detecting rare lineages and small-scale site-specific effects. However, the use of hypothesis-driven statistical testing, including *χ*^2^ goodness of fit tests against species-specific expected distributions and PERMANOVA analyses, rather than purely descriptive clustering, supports cautious but robust comparative inference of population structure between *C. coli* and *C. jejuni* across the dataset.

Genomic analysis indicated that *C. coli* often clustered by country, forming groups reflecting localized expansions in Bangladesh or Vietnam. In contrast, *C. jejuni* often showed more dispersed distributions of genetically similar isolates across countries, implying broader ecological plasticity for *C. jejuni* that spreads beyond national borders. PERMANOVA confirmed that these visual patterns were statistically robust: country of origin explained a larger amount of genomic variation in *C. coli* than in *C. jejuni*, both with cgMLST distances and with ClonalFrameML phylogenetic distances. Notably, the country effect for *C. coli* was slightly strengthened after recombination correction (cgMLST *R*²=0.146; phylogenetic *R*²=0.169), indicating that geographic clustering reflects true lineage divergence with minimal recombination events. By contrast, the country effect for *C. jejuni* decreased after recombination correction (cgMLST *R*²=0.078; phylogenetic *R*²=0.052), suggesting that recombination contributes to a greater extent to the apparent geographic similarity and that the underlying population is more mixed across countries. These patterns align with published estimates showing that recombination generates new alleles at approximately seven times the rate of point mutation in *C. jejuni*, whereas recombination and mutation contribute more equally in *C. coli* [37], potentially explaining the stronger retention of geographic structure in the latter species. PERMANOVA within-country analyses also showed that poultry type effects were more consistently detected for *C. jejuni* than *C. coli*: for *C. jejuni*, significant effects appeared in Bangladesh, Sri Lanka and Vietnam, whereas for *C. coli* only Bangladesh showed a significant effect with cgMLST distances that was not replicated with phylogenetic distances. This pattern is consistent with greater ecological plasticity in *C. jejuni*, whereby local husbandry contexts may leave a small but detectable genomic imprint. *C. jejuni* and *C. coli* can infect a broad range of hosts, with *C. jejuni* having a higher host jumping frequency, movement from one host species to another, of 1.6–1.8 years, as opposed to *C. coli* CC ST-828 clonal complex being 12 years [4]. This reduced host jumping ability may contribute to *C. coli* being more geographically restricted than *C. jejuni*, as less frequent host switching could limit opportunities for geographic dispersal via mobile hosts, such as migratory birds or international animal trade networks. In contrast to *C. coli*, *C. jejuni* was associated with multiple ST complexes that appear worldwide, suggesting more frequent inter-country transmission, possibly aided by trade activity, wild birds or other vectors [5]. As poultry production intensifies across South and Southeast Asia, *C. jejuni* may adapt to diverse niches and spread among countries and chicken production and distribution networks. This more flexible circulation was observed by clonal complexes CC ST-21, CC ST-257 and CC ST-45, emphasizing the need for regional collaboration in monitoring *C. jejuni* and *C. coli* dynamics.

The *C. coli* CC ST-828 clonal complex formed multiple sublineages tied to specific countries. For example, cluster 16 in Bangladesh was mostly ST-3236 (38%), but also contained ST-828, ST-1770 and several lesser-known variants, indicating localized genetic structure. In Vietnam, cluster 17 showed a more balanced mix of ST-1586, ST-830, ST-829 and ST-3020, suggesting that repeated introductions and gene flow contribute to population expansion in that locality. Likewise, cluster 19 (Vietnam) and cluster 22 (Sri Lanka) each showed distinct lineages such as ST-829 or ST-860, indicating further diversification among geographically defined groups. Taken together, ST data for *C. coli* provided a finer detail than clonal complex analyses alone, highlighting microdiversity that possibly reflects localized poultry breeding practices, management strategies or episodic transfers across borders.

Accessory genome analysis provided further insight, showing *C. coli* isolates clustered more distinctly by country, consistent with localized gene pools and repeated circulation within each region’s production system, while *C. jejuni* accessory gene content appeared more intermixed, consistent with broader gene flow. These patterns align with the geographic structuring demonstrated by cgMLST, ClonalFrameML and PERMANOVA analyses. However, accessory-genome PCoA requires cautious interpretation, as clustering can reflect sampling design, clonal complex composition and lineage expansion in addition to ecological processes. Both species showed pangenomes dominated by rare genes (*C. coli*, 66.9%; *C. jejuni*, 69.0%), and the first two axes captured only modest proportions of total variation. Country-level separation in *C. coli* is, therefore, consistent with this geographic structuring, rather than direct evidence of country-specific ecological adaptation. The predominance of poultry isolates in the *C. coli* dataset limited assessment of host-based patterns. For *C. jejuni*, poultry-human separation in the combined dataset was likely influenced by the inclusion of reference genomes from human sources, as the study isolates were predominantly from poultry. This pattern may be influenced by geographic and sampling differences between study and reference isolates.

This flexibility could promote rapid acquisition of novel traits, such as antimicrobial resistance or virulence genes, an important consideration for public health surveillance given the zoonotic potential of both species. If *C. coli* shows more geographically structured accessory gene variation, interaction with *C. jejuni* in poultry environments may aid acquisition of virulence or antimicrobial resistance-related genes through horizontal gene transfer and despeciation [6,41].

Patterns of cluster distribution along the farm-to-endpoint (market and slaughtering facilities) also indicated that *C. jejuni* and *C. coli* isolates travel through the supply chain. Both species, despite differing levels of geographic structuring, were detected at farms, markets and slaughtering facilities, indicating that interventions should be implemented at multiple points. For instance, certain *C. coli* clusters showed a near even split between farm and market isolates, while a sizeable subset of *C. jejuni* appeared at slaughtering facilities. These findings highlight the critical role of improved biosecurity on farms and stricter hygiene at slaughter or processing plants to minimize onward transmission to consumers, while indicating that any interventions may need tailoring to regional production systems, particularly for *C. jejuni*, where site-specific pathways may be more pronounced.

Common human-associated clonal complexes (e.g. ST-45, ST-257) were detected among local poultry populations, linking these isolates to clinical infections reported in other regions. From a global One Health perspective, lineages from poultry in specific locations have been found in other hosts, such as cattle, swine, wild birds or humans around the world. For example, CC ST-828 clonal complex has been observed in swine in Europe, and *C. jejuni* CC ST-21 complex strains have appeared in wild waterfowl. Consequently, what seems geographically confined may eventually traverse ecological boundaries through international poultry trade, migratory bird movements and/or human travel.

In the future, longitudinal studies investigating other environmental niches would help clarify how these clusters may evolve over time and whether changes in poultry management or intervention strategies (e.g. future vaccines) might affect *Campylobacter* spp. prevalence. Isolates from additional reservoirs (e.g. wild birds, cattle and swine) and from human clinical sources in South and Southeast Asia would provide a more complete view of *C. jejuni* and *C. coli* ecology, including the large discrepancy in the incidence of human infections between various *Campylobacter* spp. Likewise, continued monitoring of accessory genomes, particularly for resistance genes, could provide an early warning system for emerging high-risk strains, benefitting both animal production and public health.

## Conclusion

This study provides a baseline comparative genomic framework for *C. jejuni* and *C. coli* across poultry production networks in South and Southeast Asia. Population structure was primarily shaped by geography, with clearer phylogeographic separation observed in *C. coli* compared with *C. jejuni*. These findings support geographically targeted surveillance and species-specific risk assessment to inform intervention strategies in rapidly intensifying poultry systems.

## Supplementary material

10.1099/mgen.0.001706 Uncited Supplementary Material 1.

10.1099/mgen.0.001706 Uncited Supplementary Material 2.

## Abbreviations

CCs: clonal complexes
cgMLST: core-genome multilocus sequence typing
ESBL: extended-spectrum beta-lactamase
HICs: high-income countries
iTOL: Interactive Tree of Life
LMICs: low- and middle-income countries
LSHTM: London School of Hygiene and Tropical Medicine
ML: maximum-likelihood
MLST: multilocus sequence typing
MST: minimum spanning tree
NCBI: National Center for Biotechnology Information
PCoA: principal coordinate analysis
PERMANOVA: permutational multivariate ANOVA
STs: sequence types
WGS: whole-genome sequencing
