# Supplementary material for: Geographic clustering and population structures of Campylobacter jejuni and Campylobacter coli in South and Southeast Asian poultry systems
Source: Microb Genom. 2026 May 7;12(5):001706. doi: 10.1099/mgen.0.001706 (PMC13152252; doi:10.1099/mgen.0.001706)
Supplement: Uncited Supplementary Material 1. [file mgen-12-01706-s001.pdf]

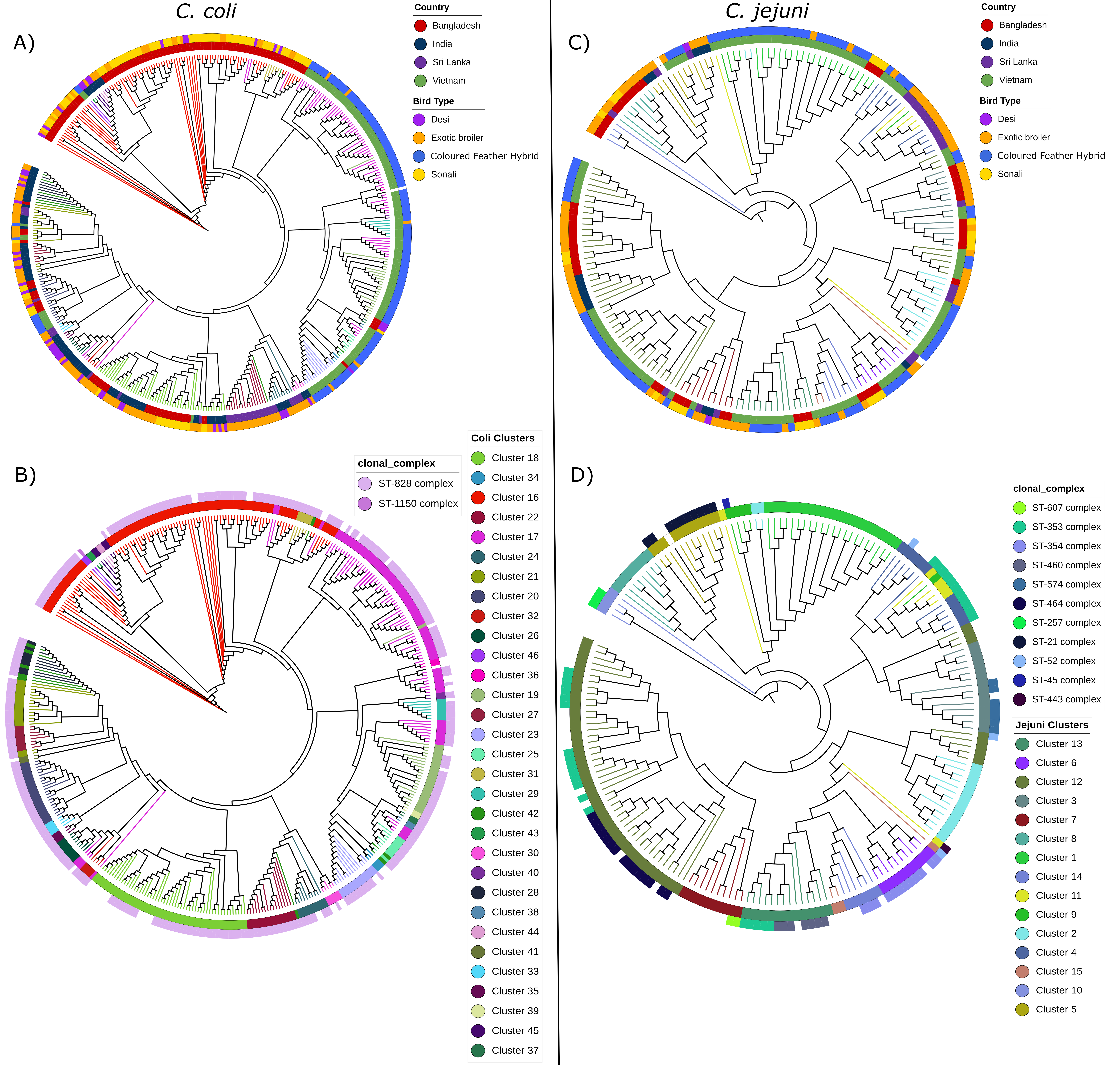

Supplementary figure S1. SNP-based radial phylogeny of *C. coli* and *C. jejuni* isolates. Radial phylogeny inferred using ClonalFrameML analysis to correct for recombination events and visualised in iTOL. (A) The two concentric rings indicate the isolates country of origin (inner ring) and chicken type (outer ring). (B) The two concentric rings represent the distribution of clonal complexes (inner ring) and clusters as determined by cgMLST (outer ring). Coloured branches highlight clusters as determined by cgMLST.

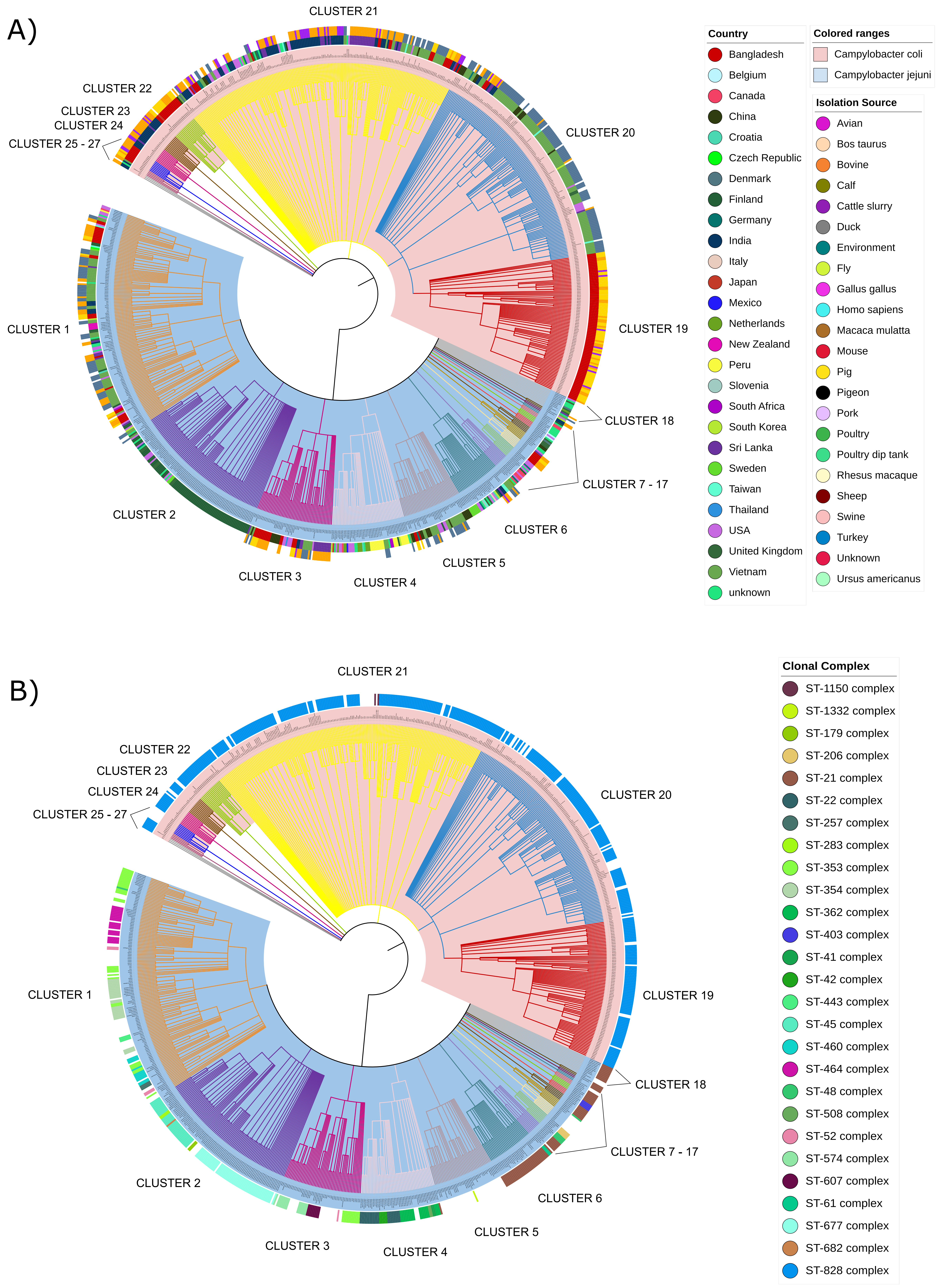

Supplementary figure S2. Radial phylogney of isolates combined with global reference strains. Radial cgMLST MST of all *C. coli* and *C. jejuni* isolates from South (India, Sri Lanka, Bangladesh) and Southeast Asia (Vietnam) combined with global curated sequences, made using chewBBACA (cgMLST) and visualised on iTOL. (A) The two concentric rings indicate country of origin (inner ring) and isolation source (outer ring). The background colours distinguish *C. coli* (pink) from *C. jejuni* (blue). Coloured branches define major clusters across the tree.

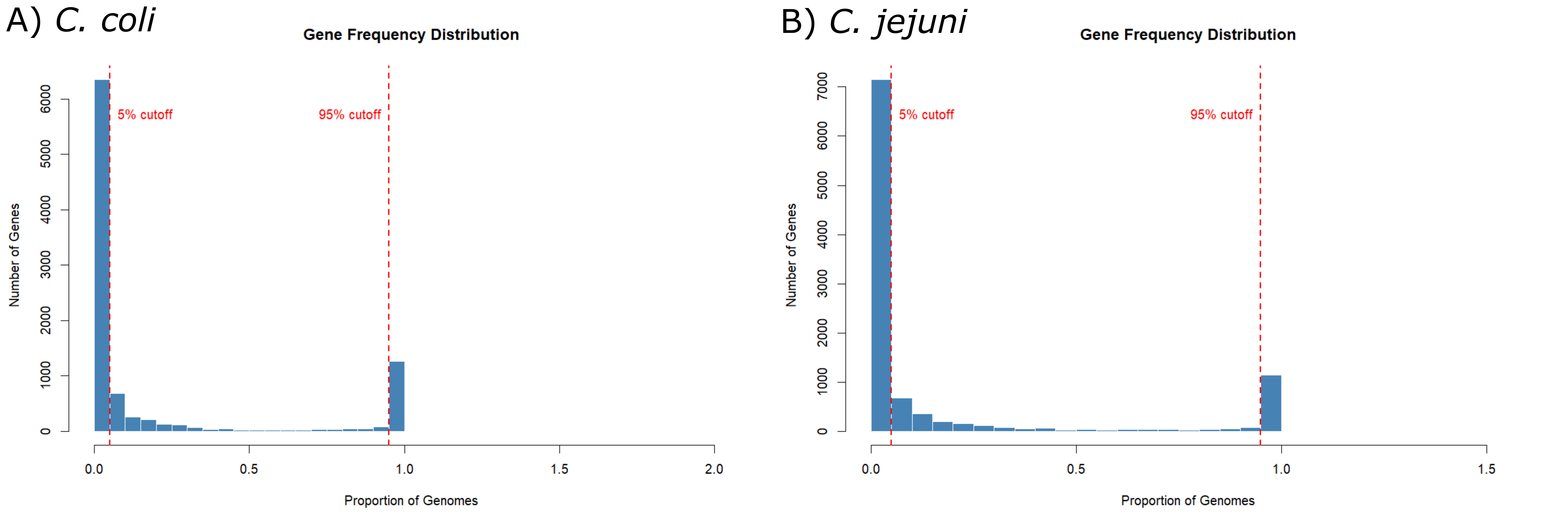

Supplementary figure S3. Frequency distribution plot for pangenome analysis. (A) *C. coli* and (B) *C. jejuni*, showing the frequency distribution of genes by the number of genomes in which each gene is found. Genes at the left end of the x-axis are rare (present in only a few genomes), while genes on the right are more prevalent (present in many genomes). Vertical red dashed lines represent the 5% and 95% prevalence.
